# Animal Research in Spain: A Study of Public Perception and Attitudes

**DOI:** 10.3390/ani13122039

**Published:** 2023-06-20

**Authors:** Andrea Miguel-Batuecas, Manuel Fuertes-Recuero, David Díaz-Regañón, Gustavo Ortiz-Díez, Luis Revuelta, Juan A. De Pablo-Moreno

**Affiliations:** 1 Department of Genetics, Physiology and Microbiology, School of Biology, Complutense University of Madrid, C/José Antonio Novais 12, 28040 Madrid, Spain; andmig01@ucm.es; 2 Department of Physiology, College of Veterinary Medicine, Complutense University of Madrid, Avda. Puerta de Hierro s/n, 28040 Madrid, Spain; manufuer@ucm.es (M.F.-R.); lrevuelt@ucm.es (L.R.); 3 Department of Animal Medicine and Surgery, College of Veterinary Medicine, Complutense University of Madrid, Avda. Puerta de Hierro s/n, 28040 Madrid, Spain; drdiazreganon@ucm.es (D.D.-R.); gusortiz@ucm.es (G.O.-D.)

**Keywords:** animal research, gender, education level, media incident, survey

## Abstract

**Simple Summary:**

Although animals are extensively used in experimentation for diverse purposes, ethical concerns have led to reluctance among a certain sector of the population. To find out the opinions of the Spanish population regarding animal research, we conducted a survey, obtaining responses from more than 800 people. While the majority were in favor, some groups were more prone to be against animal experimentation. In addition, we observed that people’s opinions could be altered by reading media reports. We also observed that people did not know about the handling of animals or laws concerning animal testing. Furthermore, we found that people were against the use of animals for non-medical purposes. In general, the survey showed the variety of people’s opinions and their concern for animal welfare.

**Abstract:**

Since the time of Hippocrates in the 4th century BC, animal research has been extensively used for various purposes up to the present day. However, the use of animals for research has also been controversial for a long time. We report the findings of a public, online questionnaire-based survey designed to assess the opinions of a sample of Spanish society regarding animal research. Demographic data and opinions were obtained from 806 respondents. The results indicated a high level of acceptance of animal research (73.1%). However, certain factors, such as completing the questionnaire immediately after a reading negative media report (OR = 2.41; 95%CI: 1.64–3.54; *p* < 0.001), being a woman (OR = 1.77; 95%CI: 1.24–2.53; *p* = 0.002) or having a non-scientific background (OR = 2.47; 95%CI: 1.76–3.47; *p* < 0.001), were associated with a tendency towards a more negative opinion. The opinions seemed to be influenced by gender, education level and by protest incidents reported in the media. Our results also indicate that a lot of information regarding animal welfare, such as care and handling protocols, along with legislation was unknown to individuals. Further, a growing popularity of companion species and opposition to animal experimentation for non-biomedical purposes were reflected in the responses obtained. The use of animals for research purposes emerged as a sensitive social issue in terms of concerns about animal ethics and welfare.

## 1. Introduction

Since the time of Hippocrates in the 4th century BC, animal research has sought to expand scientific knowledge and resulted in many advances in fields such as biomedicine [1,2]. Effectively, the many species of animals used in research [3,4] have become essential tools to understand and characterize human physiology in terms of both health and disease [5,6,7,8]. Also, an estimate of 70% of emerging infectious diseases have animal origins [9]. It is important to emphasize the relevance of animal experimentation in the study of animal diseases, especially zoonotic diseases, and its vital role in Public Health. In addition, the use of new technologies, such as Geographic Information Systems (GIS) and Remote Sensing, can be essential for their management and planning towards the One Health perspective [10,11]. However, some authors claim that animal testing is insufficient to predict clinical outcomes among humans, and they question the reliability of many animal test results. They consider many tests to be a waste of economic resources and harmful to animals [12].

In each country or region, scientific studies involving the use of animals are regulated by different laws: Directive 2010/63/EU of the European Parliament and European Council [13] in Europe, the Animal Welfare Act (AWA) [14] in the United States (protects all warm-blooded animals in research except rats, mice and birds bred), and the Animal Welfare Act [15] in the UK, along with similar regulations in other countries with developed scientific, technological systems. To try to ensure the welfare of the animals used for research purposes, all legislation is based on the principle of the 3Rs defined by Russell and Burch (Replace, Reduce and Refine) [16]. The field of alternative methods to animal experimentation has been growing and diversifying in recent years and has impacted many other disciplines. In this context, the term “alternative methods” is no longer precisely defined and has been replaced by the term, “3R methods” [17]. According to European Directive 2010/63/EU [13], all personnel working with experimental animals must be educated in how to work with animals. In many countries, courses have been established or are still in process to meet this requirement and to be educated and regularly updated on the advances being made with respect to the 3Rs. Due to the complexity of this diverse field, it is mandatory that experts share their knowledge to develop comprehensive teaching programs, gain a clear understanding of the advantages and limitations of the 3Rs approaches and how to convey them to all population target groups [18].

There are numerous limitations regarding animal experimentation such as problems with the translation and extrapolation of results to humans [19,20]. The proper selection of animal species for research is one of the main pillars for any subsequent clinical translation [21,22]. With advancing scientific knowledge and technological progress, alternative models of biomedical research are now emerging, helping to replace experimental animal models [6,23].

Owing to welfare concerns, the use of animals in research has become a controversial issue at political, social, and economic levels. National and international opinion surveys have reflected different points of view in different populations, such as healthcare professionals and veterinarians [24,25,26,27]. In a public survey conducted in Japan in 2019 [27], 51–57% of interviewees mentioned they considered animal experiments to be cruel and painful. However, 55–62% stated that animal experimentation is necessary for advances in human medicine and to guarantee the health and safety of people. In another public survey on the use of laboratory animals administered to US medical students and physicians in 2016 [25], there was widespread opposition to using animals in research based in expectations and preferences that alternative models and methods should be used.

According to an opinion survey in 2001 [26], Spanish psychology students felt that animal research was a necessary means of scientific advance. In 2021, another Spanish survey sought the opinions of people who had worked with animals in research [24]. The results indicated differing ethical concerns when experimentation concerned the use of monkeys and mice compared to that of companion animals (dogs) and farm animals (pigs).

The aim of the present study was to describe current opinions and concerns about animal research, as well as knowledge of the topic among the general Spanish population.

## 2. Materials and Methods

### 2.1. Study Design

A form that did not allow duplicate responses to be given was created on the Google Forms^®^ platform. The data collected from the replies to 21 questions were related to population demographics (age, gender, place of residence, education level and employment status), public opinion and knowledge regarding animal experimentation and alternative methods. The survey was launched online (on social media platforms) in 25 March 2021 and continued until 26 April 2021. The questionnaire was designed to obtain information or opinions regarding: agreement with the use of animals in research (Question 5), general knowledge regarding animal experimentation (Questions 6–9), the need for animal experiments for different purposes (Questions 10–13), different animal species as subjects of animal experiments (Questions 14–15), knowledge about alternative methods to animal models for use in research (Questions 16–17), required indications about the use of animal research in product development (Questions 18–18a), animal research related to SARS-CoV-2 (Questions 19–20) and the overall utility of the survey (Question 21).

While data were being collected for this study, there was an incident in which animal right activists published video images of what was supposedly animal abuse in a commercially run animal experimentation laboratory. In the remaining sections, this incident reported in the media is referred to as the “media incident”, and some respondents completed the questionnaire immediately after this report was published. The questionnaire was adequately completed by 806 anonymous voluntary respondents. The data provided were carefully revised and prepared for subsequent statistical analysis (multivariable logistic regression model). The questionnaire is provided in the Supplementary Material.

### 2.2. Data Collection

A non-probabilistic snowball sampling approach was used to recruit participants. The weblink to the online survey was accessed via institutional websites, social networks (i.e., Facebook, Twitter and Instagram) and messaging systems, such as WhatsApp, Telegram and SMS. The only information that the respondents were given at the beginning was the title of the survey before starting, a brief explanatory document stating that it was an opinion survey on animal research and a direct link to the survey. Data were collected through the online questionnaire and accessible using smart phones, tablets and personal computers.

### 2.3. Statistical Analysis

Descriptive data from the answers are expressed as ratios for categorical variables, and as the median and interquartile range (IQR) for ordinal variables. A univariate logistic regression model was used in questions (questions 5–13, 16, 17 and 18–21) comparing different responses and demographic variables according to gender (man/woman/other), age (18 to 24 and over 24 years of age), area of residence (rural/urban), region where the respondents lived (Community of Madrid/others), scientific background (yes/no), education level (non-university/ university) and knowledge regarding the controversial media incident (questionnaire completed before/after). For questions with ordinal responses, two groups were created using the median as the cut-off point. A multivariate regression model was built from the univariate regression model. Variables returning a *p* < 0.100 in the univariate regression model were considered to be relevant and included in the multivariate logistic regression analysis. The final model was built using stepwise forward selection and backward elimination techniques. The significance levels set were *p* < 0.050 for forward selection and *p* < 0.100 for backward elimination. Odds ratios (ORs) were calculated along with their 95% confidence intervals (CI). All statistical tests were performed using the software package, SPSS, version 25 (IBM Corp., Armonk, NY, USA).

## 3. Results

### 3.1. Demographics

The survey was completed by 806 persons (64.3% women, 35.1% men and 0.6% not identifying with either of the above). The most represented age group was 18–24 years (58.3%). A total of 93.8% of the participants lived in an urban area (defined as more than 30,000 inhabitants and a population density greater than 100 inhabitants per km^2^). A total of 65.4% lived within the Community of Madrid, and the remaining 31.7% were from elsewhere in the country.

When asked about education level, 73.0% reported they were undergraduates or had completed first-stage university studies or Master’s or Doctorate studies. A total of 46.5% were enrolled in a science degree course or worked in science. Most (80.9%) had completed the survey before the controversial media incident on 10 April 2021 (see Section 2). The demographic data for the respondents are provided in Table 1.

### 3.2. Questions

#### 3.2.1. Agreement with the Use of Animals in Research (Question 5)

A total of 73.1% of the respondents stated that they agreed with animal research. Among those that were more likely to say they were opposed were women, people without a scientific background and those who had completed the questionnaire just after the media incident (OR = 1.77; 95%CI: 1.24–2.53; *p* = 0.002, OR = 2.47; 95%CI: 1.76–3.47; *p* < 0.001, OR = 2.41; 95%CI: 1.64–3.54; *p* < 0.001, respectively).

#### 3.2.2. General Knowledge Regarding Animal Experimentation (Questions 6–9)

When the respondents were asked about how much animal research they thought was being conducted or how much information they had on it, the scores obtained were medians of 8 (IQR: 7–9) and 3 (IQR: 2–5), respectively. However, those with a scientific background and those responding before the media incident (OR = 3.12; 95%CI: 2.33–4.18; *p* < 0.001, OR = 1.55; 95%CI: 1.06–2.27, *p* = 0.023, respectively) were more likely to report if they were familiar with the topic of animal research.

When they were asked about legislation, 64.4% of the respondents claimed that they were aware of strict animal research regulations, but only 34.4% of the respondents had heard of the principle of the 3Rs by Russell and Burch. People with a scientific background (OR = 2.95; 95%CI: 2.08–4.20; *p* < 0.001), those who underwent university studies (OR = 2.04; 95%CI: 1.42–2.93; *p* < 0.001) and those who had responded before the media incident (OR = 2.50; 95%CI: 1.70–3.67; *p* < 0.001) were more likely to know about the existence of strict regulations. Further, respondents with a scientific background (OR = 9.43; 95%CI: 6.65–13.36; *p* < 0.001) showed a greater awareness of the principle of the 3Rs by Russell and Burch than the other participant subsets did.

#### 3.2.3. Need for Animal Experiments for Different Purposes (Questions 10–13)

When they were asked whether animal experimentation was necessary for different purposes, a large proportion of the respondents felt it was necessary for biomedical purposes (median 8; IQR: 6–10) and to address human medical issues like diseases (median 8; IQR: 6–9). However, there was wide opposition to the use of animals to test non-medical products like cosmetics (median 2; IQR: 0–5).

Respondents who were men, older than 24 years, had a science background or had responded before the media incident (OR = 1.45; 95%CI: 1.07–1.97; *p* = 0.018, OR = 1.43; 95%CI: 1.04–1.96; *p* = 0.028, OR = 2.07; 95%CI: 1.53–2.79; *p* < 0.001, OR = 1.64; 95%CI: 1.12–2.40; *p* = 0.012, respectively) were found more likely to support animal research for biomedical purposes. Further, these subsets of participants (OR = 1.53; 95%CI: 1.13–2.02; *p* = 0.006, OR = 1.48; 95%CI: 1.09–2.02; *p* = 0.013, OR = 1.73; 95%CI: 1.28–2.32; *p* < 0.001, OR = 1.79; 95%CI: 1.20–2.68; *p* = 0.005, respectively) were also more likely to be supportive of animal research to address human diseases. Additionally, men emerged as more likely to support animal testing of non-medical products than the other groups were (OR = 1.63; 95%CI: 1.22–2.19; *p* = 0.001).

A total of 84.2% of respondents agreed with the use of a drug tested in the same species as their pet. Non-residents of the Community of Madrid, those with a scientific background, those with university degrees, those who responded before the media incident (OR = 1.96; 95%CI: 1.22–3.17; *p* = 0.006, OR = 1.88; 95%CI: 1.19–2.96; *p* = 0.007, OR = 1.60; 95%CI: 1.02–2.50; *p* = 0.042, OR = 2.03; 95%CI: 1.30–3.18; *p* = 0.002, respectively) or those who had a sick pet were also more likely than the remaining groups were to agree with testing medications in species for which the drug was intended.

#### 3.2.4. Different Animal Species as Subjects of Animal Experiments (Questions 14–15)

More than three-quarters of the respondents reported that the animals most frequently used in animal research were mice (83.0%) and rats (78.5%). Only 26.6% of participants mentioned that primates were among the most used animals. Cats and fish were described as the least used ones (1% and 0.7%, respectively).

When asked which animals they felt should be used for research, 73.9% indicated they would choose mice, followed by flies, non-human primates and fish (32.6%, 26.1% and 16.6%, respectively). Only 15.1% were indifferent to the use of a given animal species for research.

#### 3.2.5. Knowledge about Alternative Methods to Animal Models for Use in Research (Questions 16 and 17)

When they were asked if they knew about alternatives to the use of animals in research, 77.8% of respondents stated there were alternatives. Compared to other demographic groups, women, those living in urban areas and those with a scientific background (OR = 1.48; 95%CI: 1.04–2.11; *p* = 0.030, OR = 3.40; 95%CI: 1.65–6.99; *p* = 0.001, OR = 2.94; 95%CI: 2.01–4.30; *p* < 0.001, respectively) were much more likely to know about the existence of alternative methods. Respondents who mentioned this were asked to describe the methods they knew about. The best-known methods were cell lines (58.4%), followed by invertebrates (22.0%), in silico models (20.9%) and organ microchip models (19.9%).

Further, when asked about this topic, 73.9% of respondents agreed that these alternative methods should be employed more often than animal models are. Women and those without a scientific background (OR = 1.79; 95%CI: 1.29–2.48; *p* < 0.001, OR = 1.40; 95%CI: 1.02–1.93; *p* = 0.04, respectively) were more likely agree with this idea.

#### 3.2.6. Require Indications about the Use of Animal Research in Product Development (Questions 18 and 18a)

The vast majority (87.2%) of participants stressed that all commercial products should clearly state whether they have been tested on animals. Of all respondents, 50.5% said they would stop using products if they did not specify whether they had been tested on animals. Women (OR = 2.71; 95%CI: 1.78–4.13; *p* < 0.001) were more likely to differ from other groups in their belief that all products must have an indication of whether they have been subjected to animal testing. In addition, the participant subsets women (OR = 2.08; 95%CI: 1.54–2.81; *p* < 0.001), those without a scientific background (OR = 1.41; 95%CI: 1.06–1.89; *p* = 0.018) and people who responded after the media incident (OR = 2.01; 95%CI: 1.38–2.92; *p* < 0.001) were more likely to state they would not continue to use a product that has been since tested on animals.

#### 3.2.7. Animal Research Related to SARS-CoV-2 (Questions 19–20)

Animal research was deemed to be essential for the study of SARS-CoV-2 by 82.3% of respondents, although 28.9% agreed it should only be used to address medical emergencies such as the COVID-19 pandemic. According to a majority of men, participants with a scientific background and those responding before the media incident, animal research was probably crucial to understanding SARS-CoV-2 and developing treatments (OR = 2.21; 95%CI: 1.44–3.41; *p* < 0.001, OR = 2.04; 95%CI: 1.39–3.00; *p* < 0.001, OR = 1.79; 95%CI: 1.17–2.76; *p* = 0.008, respectively). Individuals without a scientific background and people who completed the survey after the media event (OR = 3.27; 95%CI: 2.34–4.58; *p* < 0.001, OR = 1.92; 95%CI: 1.31–2.83; *p* = 0.001, respectively) were more likely to state that animal testing should be reserved for resolving critical issues.

#### 3.2.8. Overall Utility of the Survey (Question 21)

Among the respondents, 82.6% said that the survey had made them think about animal research. Women (OR = 2.06; 95%CI: 1.41–3.02; *p* < 0.001), respondents without a scientific background (OR = 1.89; 95%CI: 1.29–2.76; *p* = 0.001) and those who completed the questionnaire after the media incident (OR = 2.85; 95%CI: 1.52–5.33; *p* = 0.001) were more likely to say they were concerned about this.

In Table 2 and Table 3, we present all the information regarding the survey responses and their analyses, respectively. The results of our univariate test and the non-significant variables for the multivariate test are detailed in Table S1 provided in the Supplementary Material.

## 4. Discussion

Animal research generates controversy due to bioethical concerns [27,28]. This survey was designed to assess opinions about animal research in Spain. The questionnaire was completed by 806 persons. The survey tried to consider the opinions of different strata of society in Spain.

The questionnaire was designed to cover all necessary aspects when there is no specific target population. However, the main limitation of our study was that, by conducting the survey online, it is probably that fifty percent the respondents were aged 18–24 years and that only a small percentage were over 65 years. This could make the results non-extrapolatable to other age groups. We should also mention that while the initial population was largely made up of young university students living in Madrid, by making the questionnaire easily accessible to their close contacts via social media, we were able to reach other sectors of the Spanish population. Further research on this topic should ensure a targeted distribution of the questionnaire with a structural design to ensure a proper representation of the population and avoid a skewed distribution by gender, age, academic background, or even survey distribution route with an over-representation of certain sectors of the population that may limit the applicability of the results to the wider population.

Our findings revealed that over 70% of the respondents support the use of animals for research. This figure is slightly higher than that described for a given region of our country by Navarro et al. [26] in 2001, in that 65.7% of respondents strongly agreed or agreed with animal research; women and those with a non-scientific background were more likely to be against animal experimentation. Other authors also detected gender differences in the attitudes toward animal research. These differences have been explained by gender variations in socialization, attributing an emphasis on caring, nurturing and expressiveness to women rather than men [29,30,31,32]. In these studies, it was also concluded that men emphasized more the potential benefits arising from the use of animals in research [29,30,31,32]. It also seems that the general public’s lack of knowledge of the topic makes persons more sensitive to animals’ rights and suffering, leading them to question the real benefits of animal experimentation, as has been shown in other studies [33]. However, other authors suggest that these attitudes are less about a lack of knowledge and more about a perspective that comes with particular lifestyles, viewpoints and access to types of media [34]. In fact, it has been argued that as the level of awareness increases, the public may become less supportive, especially if the issue under discussion is considered to be morally contentious [35]. In contrast, people with a scientific background are likely to have a more informed opinion because of the scientific nature of the topic [32,36]. From a science communication perspective, this assumes that science is inherently the best way to acquire knowledge, and for most of the population, that is not a given fact. However, the tendency of scientifically literate people to adopt a more utilitarian perspective can, among other factors, mean that they see the necessity of using animals in research [37].

Similar to the UK study [28], our study reveals there is minimal knowledge about animal welfare regulations and animal care and handling guidelines among the Spanish public. However, it was also observed here that people feel that animal research is needed for developments in the area of human health and safety, but not for other purposes, such as testing cosmetic products. In effect, the respondents of a survey by Uchoshiki et al. [27] in 2019 reported that they approved of animal experimentation as long as it was for biomedical, and not cosmetic, purposes. In our study, a large percentage of the respondents mentioned that animal research is subject to strict regulation. This attitude was probably more common among those with a scientific background and higher education level. As seen in previous studies, having a scientific qualification and training means that respondents tend to assume they are more knowledgeable about animal research [32,36]. In Spain, animal research is regulated by Royal Decree 53/2013 [38] and the recommendations of the European Union provided in EU Directive 2010/63 [13]. These regulations are based on the principle of the 3Rs by Russell and Burch. The 3Rs are unknown to a large proportion of the general population, which focuses on the welfare of experimental animals [36], as an issue of great public concern [39]. This was also observed in the study by Iki et al. in 2017 [40], indicating a growing awareness of animal bioethics. This percentage is slightly higher than those in previous reports. This finding could be related to the characteristics of our population, with an important representation of respondents have a scientific background.

Our respondents preferred the use of mice (Mus musculus) for biomedical research above other species. This is consistent with the results of a Spanish survey performed in 2021, in which it was found that a higher percentage of people were against the use of dogs compared to those against the use of mice [24].

Motivated by the principle of the 3Rs, alternative methods have started to replace the use of animals whenever possible, and today, several of these alternative methods have roles in research [23]. Indeed, our survey showed that these models were known to most respondents. In another study [28], support for animal experimentation was linked to a lack of alternatives. This indicates respondents’ awareness of the use of alternative methods and was, in fact, evident in our survey, as a large percentage of respondents (73.9%) felt that alternatives should be used instead of experimental animals.

Another important factor that affected our results was that, at the time of our survey, an animal welfare NGO released video images reportedly taken at a pharmaceutical/biotechnological research company in Madrid [41]. The video recording depicted practices allegedly fulfilling established regulations involving verbal insults to animals and mishandling. It also showed questionable procedures performed on beagles, monkeys, mice, rabbits and pigs. That event caused an opinion bias in our respondents, in that almost twice as many people were against animal research when compared to the data obtained from forms completed earlier. As a result, animal research regulations and research for human biomedical purposes were questioned, as the video images did not seem to comply with animal welfare standards [13,38]. Despite this change in opinion, alternative methods, which are widely used in toxicological tests, have not been publicized [23]. As previously described [35], misinformation, erroneous information and the propagation of information through social networks produce alarm in some strata of society, and this leads to changing opinions or subjective opinions that are far removed from reality. It is important to emphasize that our survey indicated that the people who mentioned they had thought most about the questionnaire were those most likely to be against animal experimentation.

This study shows that, despite the fact that a large percentage of the population supports the use of research animals, it constitutes a sensitive issue about which there is a certain lack of knowledge, and it can be manifestly altered by events in a short period of time. In this sense, it is important that those responsible for formulating policies to improve those measures aimed at the transparency of the use of animals through awareness programs on this matter so that they receive impartial, unbiased information. In the same way, research facilities must always apply, in accordance with the law, measures that guarantee the animal welfare [42,43,44].

## 5. Conclusions

Our findings indicate that animal research is a sensitive social issue in Spain, mainly involving ethical and moral concerns about the use of animals in research. These concerns were sometimes motivated by a lack of information on this topic. Opinions seemed to be conditioned by gender and education level. It was also observed here that events reported in the media may cause sudden changes in public opinion. This study has highlighted the lack of information on animal experimentation provided by the scientific community to society. A potential solution to this knowledge gap could involve the integration of educational programs in countries, aimed at fostering positive attitudes and providing training to future generations regarding animal research.

## Figures and Tables

**Table 1:** Demographic data for survey participants (N = 806).

Variables		*n*	%
Age (years)			
	18–24	470	58.3
	25–29	79	9.8
	30–39	74	9.2
	40–65	176	21.8
	>65	7	0.9
Gender			
	Women	518	64.3
	Men	283	35.1
	Other	5	0.6
Living area			
	Urban	756	93.8
	Rural	34	4.2
	Not defined	16	2.0
Place of residence			
	Community of Madrid	527	65.4
	Andalusia	77	9.6
	Castilla y León	51	6.3
	Extremadura	28	3.5
	Castilla-La Mancha	25	3.1
	Valencian Community	19	2.4
	Catalonia	18	2.2
	Basque Country	17	2.1
	Outside Spain	16	2.0
	Not defined	7	0.9
	Aragon	4	0.5
	Asturias	4	0.5
	Galicia	4	0.5
	Canary Islands	4	0.5
	Balearic Islands	2	0.2
	La Rioja	2	0.2
	Navarre	1	0.1
Education level			
Non University		217	26.9
	Non-further education (including vocational training)	108	13.4
	High school	61	7.6
	Primary school	26	3.2
	Secondary school	20	2.5
	Primary education incomplete	2	0.2
University		589	73.1
	University first stage	459	56.9
	Graduate University studies (Master’s and Doctorate)	130	16.1
Scientific background			
	No	431	53.5
	Yes	375	46.5
Completion of questionnaire in relation to media incident			
	Before	652	80.9
	After	154	19.1

**Table 2:** Survey responses (Questions 5–21).

Variables		*n* (N = 806)	%
Do you approve of using animals for research purposes? (Question 5)			
	Yes	589	73.1
	No	217	26.9
How much animal research do you think is done (from 0 to 10, where 0 = none and 10 = a lot)? (Question 6)			
		8.0 *	(7.0–9.0) **
How much do you think you know about animal research e.g., protocols, legislation, etc (from 0–10, where 0 = nothing and 10 = a lot)? (Question 7)			
		3.0 *	(2.0–5.0) **
Do you think that animal research is subject to strict regulation? (Question 8)			
	No	287	35.6
	Yes	519	64.4
Do you know the principle of the 3Rs by Russell and Burch? (Question 9)			
	No	529	65.6
	Yes	277	34.4
How necessary do you think animal experimentation is for biomedical research (from 0–10, where 0 = not at all and 10 = very)? (Question 10)			
		8.0 *	(6.0–10.0) **
How much do you agree with using animals to find a cure for human diseases (from 0–10, where 0 = not at all and 10 = very much)? (Question 11)			
		8.0 *	(6.0–9.0) **
If you have or had a sick pet that needed medication, would you agree that this medication should be tested on the same species as your pet for a drug agency to approve it? (Question 12)			
	No	127	15.8
	Yes	679	84.2
How much do you agree with using animals to test non-medicinal products (from 0–10, where 0 = not at all and 10 = very much)? (Question 13)			
		2.0 *	(0.0–5.0) **
Which animals do you think are the most used in research? (Question 14)			
Mouse	No	137	17.0
	Yes	669	83.0
Rat	No	173	21.5
	Yes	633	78.5
Non-human primate	No	594	73.7
	Yes	212	26.3
Dog	No	772	95.8
	Yes	34	4.2
Rabbit	No	785	97.4
	Yes	21	2.6
Invertebrates	No	790	98.0
	Yes	16	2.0
Farm animals	No	793	98.4
	Yes	13	1.6
Cat	No	794	98.5
	Yes	12	1.5
Others	No	798	99.0
	Yes	8	1.0
Fish	No	800	99.3
	Yes	6	0.7
Which animal would you choose for biomedical research? (Question 15)			
Mouse	No	210	26.1
	Yes	596	73.9
*Drosophila melanogaster* (fruit fly)	No	543	67.4
	Yes	263	32.6
Non-human primate	No	596	73.9
	Yes	210	26.1
Fish	No	672	83.4
	Yes	134	16.6
Indifferent	No	684	84.9
	Yes	122	15.1
Dog	No	777	96.4
	Yes	29	3.6
Do you think there are alternative methods to animal models for research? (Question 16)			
	No	179	22.2
	Yes	627	77.8
If you think there are alternative methods to animal models for research, indicate the ones you know about. (Question 16a)			
In vitro cell lines	No	261	41.6
	Yes	366	58.4
Invertebrate animal models	No	489	78.0
	Yes	138	22.0
In silico analysis	No	496	79.1
	Yes	131	20.9
Tissue models: chips	No	508	81,0
	Yes	119	19.0
Do you think these alternative methods should be used more than animal models? (Question 17)			
	No	210	26.1
	Yes	596	73.9
Do you think it should be mandatory for all products to state whether animal testing was necessary for their preparation? (Question 18)			
	No	103	12.8
	Yes	703	87.2
If you answered yes, would you stop using a product that previously did not provide that information but now states it was teste in animals ? (Question 18a)			
	No	399	49.5
	Yes	407	50.5
Do you think that animal research has been or is still necessary to find a cure for COVID-19 (e.g., vaccine, treatment)? (Question 19)			
	No	143	17.7
	Yes	663	82.3
Do you think that scientists should conduct animal testing only in certain situations such as the SARS-CoV-2 emergency, and not for other diseases? (Question 20)			
	No	573	71.1
	Yes	233	28.9
Has this survey made you think about animal research? (Question 21)			
	No	140	17.4
	Yes	666	82.6

* Data expressed as median. ** Data expressed as IQR.

**Table 3:** Multivariate regression model of Questions from 5 to 13 and from 16 to 21.

**Question 5. Do you approve of using animals for research purposes?**								
**Variable**		**Participation (*n*)**		**Multivariate**				
		**Yes**	**No**	**OR**	**95%CI**			***p*-Value**
**Gender**	Men	225	58	1.00				
	Women	362	156	1.77	1.24	-	2.53	0.002
**Scientific background**	Yes	304	71	1.00				
	No	285	146	2.47	1.76	-	3.47	<0.001
**Media incident**	Before	499	153	1.00				
	After	90	64	2.41	1.64	-	3.54	<0.001
**Question 6. How much animal research do you think is done (0 = none to 10 = a lot)?**								
**Variable**		**Participation (*n*)**		**Multivariate**				
		**<median (<8.0)**	**≥median (≥8.0)**	**OR**	**95%CI**			***p*-Value**
**Gender**	Men	119	164	1.00				
	Women	181	337	1.35	1.00	-	1.82	0.047
**Question 7. How much do you think you know about animal research (e.g., protocols, legislation etc.)? (0 = nothing to 10 = a lot).**								
**Variable**		**Participation (*n*)**		**Multivariate**				
		**≤median (≤3.0)**	**>median (>3.0)**	**OR**	**95%CI**			***p*-Value**
**Scientific background**	No	302	129	1.00				
	Yes	163	212	3.12	2.33	-	4.18	<0.001
**Media incident**	After	99	55	1.00				
	Before	366	286	1.55	1.06	-	2.27	0.023
**Question 8. Do you think that animal research is subject to strict regulation?**								
**Variable**		**Participation (*n*)**		**Multivariate**				
		**No**	**Yes**	**OR**	**95%CI**			***p*-Value**
**Scientific background**	No	209	222	1.00				
	Yes	78	297	2.95	2.08	-	4.20	<0.001
**Educational level university**	No	122	95	1.00				
	Yes	165	424	2.04	1.42	-	2.93	<0.001
**Media incident**	After	78	76	1.00				
	Before	209	443	2.50	1.70	-	3.67	<0.001
**Question 9. Do you know the principle of the 3Rs by Russell and Burch?**								
**Variable**		**Participation (*n*)**		**Multivariate**				
		**No**	**Yes**	**OR**	**95%CI**			***p*-Value**
**Scientific background**	No	375	56	1.00				
	Yes	154	221	9.43	6.65	-	13.36	<0.001
**Question 10. How necessary do you think animal experimentation is for biomedical research (0 = not at all to 10 = very)?**								
**Variable**		**Participation (*n*)**		**Multivariate**				
		**<median (<8.0)**	**≥median (≥8.0)**	**OR**	**95%CI**			***p*-Value**
**Age**	18–24	215	255	1.00				
	>24	126	210	1.43	1.04	-	1.96	0.028
**Gender**	Women	233	285	1.00				
	Men	105	178	1.45	1.07	-	1.97	0.018
**Scientific background**	No	211	220	1.00				
	Yes	130	245	2.07	1.53	-	2.79	<0.001
**Media incident**	After	84	70	1.00				
	Before	257	395	1.64	1.12	-	2.40	0.012
**Question 11. How much do you agree with using animals to find a cure for human diseases (0 = not at all to 10 = very much)?**								
**Variable**		**Participation (*n*)**		**Multivariate**				
		**≤median (≤8.0)**	**>median (>8.0)**	**OR**	**95%CI**			***p*-Value**
**Age**	18–24	290	180	1.00				
	>24	171	165	1.48	1.09	-	2.02	0.013
**Gender**	Women	313	205	1.00				
	Men	143	140	1.53	1.13	-	2.02	0.006
**Scientific background**	No	265	166	1.00				
	Yes	196	179	1.73	1.28	-	2.32	<0.001
**Media incident**	After	109	45	1.00				
	Before	352	300	1.79	1.2	-	2.68	0.005
**Question 12. If you have or had a sick pet that needed medication, would you agree that this medication should be tested on the same species as your pet for a drug agency to approve it?**								
**Variable**		**Participation (*n*)**		**Multivariate**				
		**No**	**Yes**	**OR**	**95%CI**			***p*-Value**
**Scientific background**	No	88	343	1.00				
	Yes	39	336	1.88	1.19	-	2.96	0.007
**Autonomous Community**	Madrid	100	427	1.00				
	Other communities	25	229	1.96	1.22	-	3.17	0.006
**Educational level university**	No	51	166	1.00				
	Yes	76	513	1.60	1.02	-	2.50	0.042
**Media incident**	After	38	116	1.00				
	Before	89	563	2.03	1.30	-	3.18	0.002
**Question 13. How much do you agree with using animals to test non-medicinal products (0 = not at all to 10 = very much)?**								
**Variable**		**Participation (*n*)**		**Multivariate**				
		**≤median (≤2.0)**	**>median (>2.0)**	**OR**	**95%CI**			***p*-Value**
**Gender**	Women	301	217	1.00				
	Men	130	153	1.63	1.22	-	2.19	0.001
**Question 16. Do you think there are alternative methods to animal models for research?**								
**Variable**		**Participation (*n*)**		**Multivariate**				
		**No**	**Yes**	**OR**	**95%CI**			***p*-Value**
**Gender**	Men	80	203	1.00				
	Women	99	419	1.48	1.04	-	2.11	0.03
**Place of residence**	Rural	17	17	1.00				
	Urban	158	598	3.40	1.65	-	6.99	0.001
**Scientific background**	No	133	298	1.00				
	Yes	46	329	2.94	2.01	-	4.30	<0.001
**Question 17. Do you think these alternative methods should be used more than animal models?**								
**Variable**		**Participation (*n*)**		**Multivariate**				
		**No**	**Yes**	**OR**	**95%CI**			***p*-Value**
**Gender**	Men	94	189	1.00				
	Women	116	402	1.79	1.29	-	2.48	<0.001
**Scientific background**	Yes	108	267	1.00				
	No	102	329	1.40	1.02	-	1.93	0.04
**Question 18. Do you think it should be mandatory for all products to state whether animal testing was necessary for their preparation?**								
**Variable**		**Participation (*n*)**		**Multivariate**				
		**No**	**Yes**	**OR**	**95%CI**			***p*-Value**
**Gender**	Men	58	225	1.00				
	Women	45	473	2.71	1.78	-	4.13	<0.001
**Question 18a. If you answered yes, would you stop using a product that previously did not provide that information but now states it was tested in animals?**								
**Variable**		**Participation (*n*)**		**Multivariate**				
		**No**	**Yes**	**OR**	**95%CI**			***p*-Value**
**Gender**	Men	173	110	1.00				
	Women	224	294	2.08	1.54	-	2.81	<0.001
**Scientific background**	Yes	198	177	1.00				
	No	201	230	1.41	1.06	-	1.89	0.018
**Media incident**	Before	345	307	1.00				
	After	54	100	2.01	1.38	-	2.92	<0.001
**Question 19. Do you think that animal research has been or is still necessary to find a cure for COVID-19 (e.g., vaccine, treatment)**								
**Variable**		**Participation (*n*)**		**Multivariate**				
		**No**	**Yes**	**OR**	**95%CI**			***p*-Value**
**Gender**	Women	110	408	1.00				
	Men	32	251	2.21	1.44	-	3.41	<0.001
**Scientific background**	No	94	337	1.00				
	Yes	49	326	2.04	1.39	-	3.00	<0.001
**Media incident**	After	40	114	1.00				
	Before	103	549	1.79	1.17	-	2.76	0.008
**Question 20. Do you think that scientists should conduct animal testing only in certain situations such as the SARS-CoV-2 emergency, and not for other diseases?**								
**Variable**		**Participation (*n*)**		**Multivariate**				
		**No**	**Yes**	**OR**	**95%CI**			***p*-Value**
**Scientific background**	Yes	311	64	1.00				
	No	262	169	3.27	2.34	-	4.58	<0.001
**Media incident**	Before	478	174	1.00				
	After	95	59	1.92	1.31	-	2.83	0.001
**Question 21. Has this survey made you think about animal research?**								
**Variable**		**Participation (*n*)**		**Multivariate**				
		**No**	**Yes**	**OR**	**95%CI**			***p*-Value**
**Gender**	Men	68	215	1.00				
	Women	71	447	2.06	1.41	-	3.02	<0.001
**Scientific background**	Yes	80	295	1.00				
	No	60	371	1.89	1.29	-	2.76	0.001
**Media incident**	Before	128	524	1.00				
	After	12	142	2.85	1.52	-	5.33	0.001

## Data Availability

The original contributions are included in the article/Supplementary Material, further inquiries can be directed to the corresponding author.

## Supplementary Materials

The following supporting information can be downloaded at: https://www.mdpi.com/article/10.3390/ani13122039/s1, Survey; Table S1. Univariate and multivariate regression model of questions 5 to 13 and 16 to 21.
