# Supplementary material for: Animal Research in Spain: A Study of Public Perception and Attitudes
Source: Animals (Basel). 2023 Jun 20;13(12):2039. doi: 10.3390/ani13122039 (PMC10294839; doi:10.3390/ani13122039)
Supplement: Supplementary file 1 [file animals-13-02039-s001.zip › animals-2421924-supplementary.pdf]

Supplementary material

Survey

Demographic data

1. Age (years)

   18-24

   25-29

   30-39

   40-65

   > 65

2. Gender

   Women

   Men

   I do not identify with either of the above

3. Place of residence

4. Education level

Primary education incomplete

Primary school

Secondary school

High school

Non-further education (including vocational training)

University first stage

Graduate University studies (Master´s, Doctorate)

a. If you are a non-university level, university first stage, Masters or PhD student in the field of Science, indicate the degree programme.

b. If you work in Science, briefly indicate where and what you do.

Public opinion data

5. Do you approve of using animals for research purposes?

Yes

No

6. How much animal research do you think is done (from 0 to 10, where 0=none and 10=a lot)?

7. How much do you think you know about animal research e.g., protocols, legislation, etc) (from 0-10, where 0=none and 10=a lot)?

8. Do you think that animal research is subject to strict regulation? Yes/No

9. Do you know the principle of the 3Rs by Russell and Burch? Yes/No

10. How necessary do you think animal experimentation is for biomedical research (from 0-10, where 0= not at all and 10= very)?

11. How much do you agree with using animals to find a cure for human diseases (from 0-10, where 0= not at all and 10= very much)?

12. If you have or had a sick pet that needed medication, would you agree that this medication should be tested on the same species as your pet for a drug agency to approve it? Yes/No

13. How much do you agree with using animals to test non-medicinal products (from 0-10, where 0=not at all and 10= very much).

Experimental animal data

14. Which animals do you think are the most used in research? * Select two options*

Dog

Mouse

Cat

Rat

Non-human primate

Other (add option)

15. Which animal would you choose for biomedical research? * Select two options *

Drosophila melanogaster (fruit fly)

Mouse

Zebra-fish

Non-human primates

Dog

Indifferent

16. Do you think there are alternative methods to animal models for research? Yes/No

16a. If you think there are alternative methods to animal models for research, indicate the ones you know about:

In vitro cell lines

Invertebrate animal models

Tissue models: chips

In silico analysis (computer simulations)

17. Do you think these alternative methods should be used more than animal models? Yes/No

18. Do you think it should be mandatory for all products to state whether animal testing was necessary for their preparation? Yes/No

18.a If you answered yes, would you stop using a product that previously did not provide that information but now states it was teste in animals? Yes/No

19. Do you think that animal research has been or is still necessary to find a cure for COVID-19 (e.g., vaccine, treatment)? Yes/No

20. Do you think that scientists should conduct animal testing only in certain situations such as the SARS-Cov-2 emergency, and not for other diseases? Yes/No

21. Has this survey made you think about animal research? Yes/No

Table S1. Univariate and multivariate regression model of questions 5 to 13 and 16 to 21.

Question 5. Do you approve of using animals for research purposes?

Variable		Participation (n)		Univariate			Multivariate		
		Yes	No	OR	95%CI	p-Value	OR	95%CI	p-Value
**Age**									
	>24	259	77		1.00			-	
	18-24	330	140	1.43	1.03 - 1.97	0.031		-	
**Gender**									
	Men	225	58		1.00			1.00	
	Women	362	156	1.67	1.18 - 2.36	0.003	1.77	1.24 - 2.53	0.002
**Living area**									
	Urban	555	201		1.00			-	
	Rural	22	12	1.51	0.73 - 3.10	0.266		-	
**Scientific background**									
	Yes	304	71		1.00			1.00	
	No	285	146	2.19	1.58 - 3.04	<0.001	2.47	1.76 - 3.47	<0.001
**Autonomous Community**									
	Other communities	189	65		1.00			-	
	Madrid	381	146	1.11	0.79 - 1.57	0.533		-	
**Education level university**									
	Yes	447	142		1.00			-	
	No	142	75	1.66	1.19 - 2.33	0.003		-	
**Media incident**									
	Before	499	153		1.00			1.00	
	After	90	64	2.32	1.60 - 3.35	<0.001	2.41	1.64 - 3.54	<0.001

Question 6. How much animal research do you think is done (0=none to 10=a lot)?

Variable		Participation (n)		Univariate			Multivariate		
		< median (< 8.0)	≥ median (≥ 8.0)	OR	95%CI	p-Value	OR	95%CI	p-Value
**Age**									
	>24	126	210		1.00			-	
	18-24	176	294	1.00	0.75 - 1.34	0.988		-	
**Gender**									
	Men	119	164		1.00			1.00	

	Women	181	337	1.35	1.00	-	1.82	0.047	1.35	1.00	-	1.82	0.047
**Living area**													
	Rural	15	19			1.00					-		
	Urban	277	479	1.37	0.68	-	2.73	0.379			-		
**Scientific background**													
	No	164	267			1.00					-		
	Yes	138	237	1.05	0.79	-	1.40	0.710			-		
**Autonomous Community**													
	Other communities	100	154			1.00					-		
	Madrid	194	333	1.11	0.82	-	1.52	0.490			-		
**Education level university**													
	Yes	226	363			1.00					-		
	No	76	141	1.16	0.83	-	1.60	0.384			-		
**Media incident**													
	After	58	96			1.00					-		
	Before	244	408	1.01	0.70	-	1.45	0.956			-		

Question 7. How much do you think you know about animal research (e.g., protocols, legislation etc.)? (0=nothing to 10=a lot).

Variable		Participation (n)		Univariate					Multivariate				
		≤ median (≤ 3.0)	> median (> 3.0)	OR	95%CI			p-Value	OR	95%CI			p-Value
**Age**													
	18-24	274	196			1.00					-		
	>24	191	145	1.06	0.80	-	1.41	0.680			-		
**Gender**													
	Women	309	209			1.00					-		
	Men	155	128	1.22	0.91	-	1.64	0.180			-		
**Living area**													
	Rural	20	14			1.00					-		
	Urban	435	321	1.05	0.52	-	2.12	0.882			-		
**Scientific background**													
	No	302	129			1.00					1.00		
	Yes	163	212	3.04	2.28	-	4.07	<0.001	3.12	2.33	-	4.18	<0.001
**Autonomous Community**													
	Other communities	148	106			1.00					-		
	Madrid	300	227	1.06	0.78	-	1.43	0.722			-		

**Education level university**									
	No	145	72		1.00			-	
	Yes	320	269	1.69	1.22 - 2.34	0.002		-	
**Media incident**									
	After	99	55		1.00			1.00	
	Before	366	286	1.41	0.98 - 2.02	0.066	1.55	1.06 - 2.27	0.023

Question 8. Do you think that animal research is subject to strict regulation?

Variable		Participation (n)		Univariate			Multivariate		
		No	Yes	OR	95%CI	p-Value	OR	95%CI	p-Value
**Age**									
	18-24	177	293		1.00			-	
	>24	110	226	1.24	1.67 - 0.92	0.150		-	
**Gender**									
	Women	189	329		1.00			-	
	Men	96	187	1.12	1.52 - 0.83	0.47		-	
**Living area**									
	Urban	270	486		1.00			-	
	Rural	11	23	1.16	0.56 - 2.42	0.689		-	
**Scientific background**									
	No	209	222		1.00			1.00	
	Yes	78	297	3.58	2.62 - 4.90	<0.001	2.95	2.08 - 4.20	<0.001
**Autonomous Community**									
	Madrid	195	332		1.00			-	
	Other communities	79	175	1.30	1.79 - 0.95	0.106		-	
**Education level university**									
	No	122	95		1.00			1.00	
	Yes	165	424	3.30	2.39 - 4.56	<0.001	2.04	1.42 - 2.93	<0.001
**Media incident**									
	After	78	76		1.00			1.00	
	Before	209	443	1.25	1.52 - 3.11	0.264	2.50	1.70 - 3.67	<0.001

Question 9. Do you know the principle of the 3Rs by Russel and Burch?

Variable		Participation (n)		Univariate			Multivariate		
		No	Yes	OR	95%CI	p-Value	OR	95%CI	p-Value
**Age**									

	>24	243	93		1.00			-	
	18-24	286	184	1.68	1.24 - 2.27	0.001		-	
**Gender**									
	Men	190	93		1.00			-	
	Women	337	181	1.10	0.81 - 1.49	0.553		-	
**Living area**									
	Rural	26	8		1.00			-	
	Urban	489	267	1.77	0.79 - 3.97	0.160		-	
**Scientific background**									
	No	375	56		1.00			1.00	
	Yes	154	221	9.61	6.78 - 13.61	<0.001	9.43	6.65 - 13.36	<0.001
**Autonomous Community**									
	Madrid	342	185		1.00			-	
	Other communities	163	91	1.03	0.75 - 1.41	0.843		-	
**Education level university**									
	No	185	32		1.00			-	
	Yes	344	245	4.12	2.73 - 6.20	<0.001		-	
**Media incident**									
	After	103	51		1.00			-	
	Before	426	226	2.17	0.74 - 1.55	<0.001		-	

Question 10. How necessary do you think animal experimentation is for biomedical research (0= not at all to 10= very)?

Variable		Participation (n)		Univariate			Multivariate		
		< median (< 8.0)	≥ median (≥ 8.0)	OR	95%CI	p-Value	OR	95%CI	p-Value
**Age**									
	18-24	215	255		1.00			1.00	
	>24	126	210	1.41	1.06 - 1.87	0.020	1.43	1.04 - 1.96	0.028
**Gender**									
	Women	233	285		1.00			1.00	
	Men	105	178	1.39	1.03 - 1.86	0.030	1.45	1.07 - 1.97	0.018
**Living area**									
	Rural	15	19		1.00			-	
	Urban	315	441	1.11	0.55 - 2.21	0.777		-	
**Scientific background**									
	No	211	220		1.00			1.00	
	Yes	130	245	1.81	1.36 - 2.40	<0.001	2.07	1.53 - 2.79	<0.001

**Autonomous Community**									
	Madrid	223	304		1.00			-	
	Other communities	103	151	1.08	0.79 - 1.46	0.640		-	
**Education level university**									
	No	106	111		1.00			-	
	Yes	235	354	1.44	1.05 - 1.97	0.020		-	
**Media incident**									
	After	84	70		1.00			1.00	
	Before	257	395	1.84	1.17 - 2.06	0.001	1.64	1.12 - 2.40	0.012

Question 11. How much do you agree with using animals to find a cure for human diseases (0= not at all to 10= very much)?

**Variable**		**Participation (n)**		**Univariate**			**Multivariate**		
		**≤ median (≤8.0)**	**> median (> 8.0)**	**OR**	**95%CI**	**p-Value**	**OR**	**95%CI**	**p-Value**
**Age**									
	18-24	290	180		1.00			1.00	
	>24	171	165	1.55	1.12 - 2.00	<0.001	1.48	1.09 - 2.02	0.013
**Gender**									
	Women	313	205		1.00			1.00	
	Men	143	140	1.49	1.04 - 2.65	0.010	1.53	1.13 - 2.02	0.006
**Living area**									
	Urban	434	322		1.00			-	
	Rural	18	16	1.20	0.60 - 2.39	0.610		-	
**Scientific background**									
	No	265	166		1.00			1.00	
	Yes	196	179	1.46	1.10 - 1.93	0.010	1.73	1.28 - 2.32	<0.001
**Autonomous Community**									
	Other communities	147	107		1.00			-	
	Madrid	302	225	1.02	0.76 - 1.39	0.880		-	
**Education level university**									
	No	134	83		1.00			-	
	Yes	327	262	1.29	0.94 - 1.78	0.110		-	
**Media incident**									
	After	109	45		1.00			1.00	
	Before	352	300	2.06	1.41 - 3.02	<0.001	1.79	1.2 - 2.68	0.005

Question 12. If you have or had a sick pet that needed medication, would you agree that this medication should be tested on the same species as your pet for a drug agency to approve it?

Variable		Participation (n)		Univariate			Multivariate		
		No	Yes	OR	95%CI	p-Value	OR	95%CI	p-Value
**Age**									
	18-24	85	385		1.00			-	
	>24	42	294	1.55	1.04 - 2.30	0.030		-	
**Gender**									
	Women	88	430		1.00			-	
	Men	39	244	1.28	0.85 - 1.93	0.240		-	
**Living area**									
	Rural	6	28		1.00			-	
	Urban	120	636	1.14	0.46 - 2.80	0.782		-	
**Scientific background**									
	No	88	343		1.00			1.00	
	Yes	39	336	2.21	1.47 - 3.32	<0.001	1.88	1.19 - 2.96	0.007
**Autonomous Community**									
	Madrid	100	427		1.00			1.00	
	Other communities	25	229	2.15	1.35 - 3.42	0.001	1.96	1.22 - 3.17	0.006
**Education level university**									
	No	51	166		1.00			1.00	
	Yes	76	513	2.07	1.40 - 3.08	<0.001	1.60	1.02 - 2.50	0.042
**Media incident**									
	After	38	116		1.00			1.00	
	Before	89	563	2.07	1.35 - 3.18	0.001	2.03	1.30 - 3.18	0.002

Question 13. How much do you agree with using animals to test non-medicinal products (0=not at all to 10= very much)?

Variable		Participation (n)		Univariate			Multivariate		
		≤ median (≤2.0)	> median (> 2.0)	OR	95%CI	p-Value	OR	95%CI	p-Value
**Age**									
	18-24	262	208		1.00			-	
	>24	174	162	1.17	0.89 - 1.55	0.270		-	
**Gender**									
	Women	301	217		1.00			1.00	
	Men	130	153	1.63	1.22 - 2.19	<0.001	1.63	1.22 - 2.19	0.001
**Living area**									
	Rural	19	15		1.00			-	
	Urban	410	346	1.07	0.54 - 2.14	0.850		-	

**Scientific background**									
	Yes	205	170		1.00			-	
	No	231	200	1.04	0.79 - 1.38	0.761		-	
**Autonomous Community**									
	Madrid	288	239		1.00			-	
	Other communities	138	116	1.01	0.75 - 1.37	0.933		-	
**Education level university**									
	Yes	327	262		1.00			-	
	No	109	108	1.24	0.91 - 1.69	0.182		-	
**Media incident**									
	After	94	60		1.00			-	
	Before	342	310	1.42	0.99 - 2.03	0.055		-	

Question 16. Do you think there are alternative methods to animal models for research?

**Variable**		**Participation (n)**		**Univariate**			**Multivariate**		
		**No**	**Yes**	**OR**	**95%CI**	**p-Value**	**OR**	**95%CI**	**p-Value**
**Age**									
	>24	45	291		1.00			-	
	18-24	58	412	1.76	1.26 - 2.46	<0.001		-	
**Gender**									
	Men	80	203		1.00			1.00	
	Women	99	419	1.67	1.19 - 2.34	<0.001	1.48	1.04 - 2.11	0.03
**Living area**									
	Rural	17	17		1.00			1.00	
	Urban	158	598	3.78	1.89 - 7.58	<0.001	3.40	1.65 - 6.99	0.001
**Scientific background**									
	No	133	298		1.00			1.00	
	Yes	46	329	3.19	2.20 - 4.62	<0.001	2.94	2.01 - 4.30	<0.001
**Autonomous Community**									
	Other communities	59	195		1.00			-	
	Madrid	114	413	1.10	0.77 - 1.57	0.610		-	
**Education level university**									
	No	67	150		1.00			-	
	Yes	112	477	1.90	1.34 - 2.71	<0.001		-	
**Media incident**									

	Before	151	501			1.00					-			
	After	28	126	1.36	0.87	-	2.12	0.180			-			

Question 17. Do you think these alternative methods should be used more than animal models?

Variable		Participation (n)		Univariate					Multivariate					
		No	Yes	OR	95%CI			p-Value	OR	95%CI				p-Value
**Age**														
	>24	92	244			1.00					-			
	18-24	118	352	1.12	0.82	-	1.55	0.470			-			
**Gender**														
	Men	94	189			1.00					1.00			
	Women	116	402	1.72	1.25	-	2.38	<0.001	1.79	1.29	-	2.48		<0.001
**Living area**														
	Rural	10	24			1.00					-			
	Urban	198	558	1.17	0.55	-	2.50	0.677			-			
**Scientific background**														
	Yes	108	267			1.00					1.00			
	No	102	329	1.30	1.79	-	0.95	0.098	1.40	1.02	-	1.93		0.04
**Autonomous Community**														
	Other communities	69	185			1.00					-			
	Madrid	135	392	1.08	0.77	-	1.52	0.640			-			
**Education level university**														
	Yes	163	426			1.00					-			
	No	47	170	1.38	0.96	-	2.00	0.085			-			
**Media incident**														
	Before	177	475			1.00					-			
	After	33	121	1.37	0.90	-	2.08	0.150			-			

Question 18. Do you think it should be mandatory for all products to state whether animal testing was necessary for their preparation?

Variable		Participation (n)		Univariate					Multivariate					
		No	Yes	OR	95%CI			p-Value	OR	95%CI				p-Value
**Age**														
	>24	45	291			1.00					-			
	18-24	58	412	1.10	0.72	-	1.67	0.659			-			
**Gender**														
	Men	58	225			1.00					1.00			
	Women	45	473	2.71	1.78	-	4.13	<0.001	2.71	1.78	-	4.13		<0.001

**Living area**									
	Rural	6	28	1.00			-		
	Urban	97	659	1.46	0.59 - 3.61	0.417	-		
**Scientific background**									
	No	62	369	1.00			-		
	Yes	41	334	1.37	0.90 - 2.09	0.140	-		
**Autonomous Community**									
	Other communities	35	219	1.000			-		
	Madrid	66	461	1.12	0.72 - 1.73	0.620	-		
**Education level university**									
	No	30	187	1.00			-		
	Yes	73	516	1.13	0.72 - 1.79	0.590	-		
**Media incident**									
	Before	89	563	1.00			-		
	After	14	140	1.58	0.87 - 2.86	0.130	-		

Question 18a. If you answered yes, would you stop using a product that previously did not provide that information but now states it was tested in animals?

Variable		Participation (n)		Univariate			Multivariate		
		No	Yes	OR	95%CI	p-Value	OR	95%CI	p-Value
**Age**									
	>24	185	151	1.00			-		
	18-24	214	256	1.47	1.11 - 1.94	0.010	-		
**Gender**									
	Men	173	110	1.00			1.00		
	Women	224	294	2.06	1.54 - 2.77	<0.001	2.08	1.54 - 2.81	<0.001
**Living area**									
	Rural	20	14	1.00			-		
	Urban	370	386	1.49	0.74 - 2.99	0.262	-		
**Scientific background**									
	Yes	198	177	1.00			1.00		
	No	201	230	1.28	0.97 - 1.69	0.081	1.41	1.06 - 1.89	0.018
**Autonomous Community**									
	Other communities	126	128	1.00			-		
	Madrid	256	271	1.04	0.77 - 1.41	0.790	-		
**Education level university**									

	Yes	292	297		1.00			-	
	No	107	110	1.01	0.74 - 1.38	0.946		-	
**Media incident**									
	Before	345	307		1.00			1.00	
	After	54	100	2.08	1.44 - 3.00	<0.001	2.01	1.38 - 2.92	<0.001

Question 19. Do you think that animal research has been or is still necessary to find a cure for COVID-19 (e.g., vaccine, treatment)

**Variable**		**Participation (n)**		**Univariate**			**Multivariate**		
		**No**	**Yes**	**OR**	**95%CI**	**p-Value**	**OR**	**95%CI**	**p-Value**
**Age**									
	18-24	90	380		1.00			-	
	>24	53	283	1.26	0.87 - 1.84	0.220		-	
**Gender**									
	Women	110	408		1.00			1.00	
	Men	32	251	2.11	1.38 - 3.23	0.001	2.21	1.44 - 3.41	<0.001
**Living area**									
	Urban	135	621		1.00			-	
	Rural	6	28	1.01	0.41 - 2.50	0.980		-	
**Scientific background**									
	No	94	337		1.00			1.00	
	Yes	49	326	1.86	1.27 - 2.71	<0.001	2.04	1.39 - 3.00	<0.001
**Autonomous Community**									
	Madrid	95	432		1.00			-	
	Other communities	45	209	1.02	0.69 - 1.51	0.916		-	
**Education level university**									
	No	50	167		1.00			-	
	Yes	93	496	1.60	1.09 - 2.35	0.020		-	
**Media incident**									
	After	40	114		1.00			1.00	
	Before	103	549	1.87	1.23 - 2.84	0.003	1.79	1.17 - 2.76	0.008

Question 20. Do you think that scientists should conduct animal testing only in certain situations such as the SARS-Cov-2 emergency, and not for other diseases?

**Variable**		**Participation (n)**		**Univariate**			**Multivariate**		
		**No**	**Yes**	**OR**	**95%CI**	**p-Value**	**OR**	**95%CI**	**p-Value**
**Age**									
	18-24	335	135		1.00			-	
	>24	238	98	1.02	0.75 - 1.39	0.890		-	

**Gender**									
	Women	372	146		1.00			-	
	Men	196	87	1.13	0.82 - 1.55	0.450		-	
**Living area**									
	Urban	545	211		1.00			-	
	Rural	18	16	2.30	1.15 - 4.59	0.020		-	
**Scientific background**									
	Yes	311	64		1.00			1.00	
	No	262	169	3.13	2.25 - 4.37	<0.001	3.27	2.34 - 4.58	<0.001
**Autonomous Community**									
	Other communities	181	73		1.00			-	
	Madrid	372	155	1.03	0.74 - 1.44	0.850		-	
**Education level university**									
	Yes	440	149		1.00			-	
	No	133	84	1.87	1.34 - 2.60	<0.001		-	
**Media incident**									
	Before	478	174		1.00			1.00	
	After	95	59	1.71	1.18 - 2.47	<0.001	1.92	1.31 - 2.83	0.001

Question 21. Has this survey made you think about animal research?

**Variable**		**Participation (n)**		**Univariate**			**Multivariate**		
		**No**	**Yes**	**OR**	**95%CI**	**p-Value**	**OR**	**95%CI**	**p-Value**
**Age**									
	>24	68	268		1.00			-	
	18-24	72	398	1.40	0.97 - 2.02	0.070		-	
**Gender**									
	Men	68	215		1.00			1.00	
	Women	71	447	1.99	1.38 - 2.88	<0.001	2.06	1.41 - 3.02	<0.001
**Living area**									
	Rural	7	27		1.00			-	
	Urban	132	624	1.23	0.52 - 2.87	0.640		-	
**Scientific background**									
	Yes	80	295		1.00			1.00	
	No	60	371	1.68	1.16 - 2.42	0.006	1.89	1.29 - 2.76	0.001
**Autonomous Community**									
	Madrid	96	431		1.00			-	

	Other communities	39	215	1.23	0.82	-	1.84	0.322			-		
**Education level university**													
	Yes	113	476			1.00					-		
	No	27	190	1.67	1.06	-	2.63	0.026			-		
**Media incident**													
	Before	128	524			1.00					1.00		
	After	12	142	2.89	1.55	-	5.37	<0.001	2.85	1.52	-	5.33	0.001
